# Patients’ experiences of physical activity on prescription with access to counsellors in routine care: a qualitative study in Sweden

**DOI:** 10.1186/s12889-019-6535-5

**Published:** 2019-02-20

**Authors:** Pia Andersen, Lena Lendahls, Sara Holmberg, Per Nilsen

**Affiliations:** 1 Department of Research and Development, Region Kronoberg, Växjö, Sweden; 2 Department of Medical and Health Sciences, Division of Community Medicine, Linköping University, Linköping, Sweden; 3 Faculty of Health and Life Sciences, Department of Health and Caring Sciences, Linnaeus University, Kalmar, Sweden; 4 Division of Occupational and Environmental Medicine, Institute of Laboratory Medicine, Lund University, Lund, Sweden

**Keywords:** Qualitative research, Physical activity, Health care, Patients, Counselling

## Abstract

**Background:**

Physical activity on prescription (PAP) has been implemented in several countries, including Sweden, to support patients who might benefit from increased physical activity. This study explores the experiences of recipients of PAP in routine health care in Sweden that offers the recipients support from physical activity counsellors. The aim was to explore influences on engagement in physical activity by PAP recipients’ from a long-term perspective.

**Methods:**

We conducted individual semi-structured interviews using a topic guide with a purposively selected sample of 13 adult PAP recipients 1.5 to 2.5 years after PAP. Interviews were recorded, transcribed verbatim and analysed through inductive and deductive content analysis. The questions were informed by Capability-Opportunity-Motivation-Behaviour (COM-B), which was also used as a framework to analyse the data by means of categorizing the factors (influences on the behaviour).

**Results:**

Ten factors (i.e. sub-categories) that influenced the participants’ engagement in physical activity were identified. PAP recipients’ capability to engage in physical activity was associated with adapting the PAP to the individual’s physical capacity and taking into account the individual’s previous experiences of physical activity. PAP recipients’ opportunity to engage in physical activity was related to receiving a prescription, receiving professional counselling and follow-up from a physical activity counsellor, collaboration between prescriber and counsellor, having access to appropriate activities, having a balanced life situation and having support from someone who encouraged continued physical activity. PAP recipients’ motivation to engage in physical activity was associated with the desire to improve his or her health condition and finding activities that encouraged continuation.

**Conclusions:**

PAP recipients’ engagement in physical activity was influenced by their capability, opportunity and motivation to undertake this behaviour. Numerous extraneous factors influence capability and motivation. Physical activity counsellors were found to be important for sustained activity because they use an individual approach to counselling and flexible follow-up adapted to each individual’s need of support.

**Electronic supplementary material:**

The online version of this article (10.1186/s12889-019-6535-5) contains supplementary material, which is available to authorized users.

## Background

There is convincing evidence supporting the role of physical activity in the prevention and treatment of non-communicable diseases. Globally, one in four adults report physical activity below recommended levels, and the proportion is even higher in Sweden: one in three adults [1]. Numerous barriers have been identified for adult participation in physical activity, such as lack of social support [2, 3], insufficient motivation and unfavourable health status [4–6]. Physical activity on prescription (PAP) in routine health care has been implemented in several countries, including Sweden, to support patients who might benefit from increased physical activity [7]. These programmes vary with regard to design, e.g. programme duration, reason for prescription and patient payments. Varying degrees of effectiveness in influencing patients’ physical activity have been observed [7]. It is important that PAP programmes enhance recipients’ self-efficacy to participate in physical activity [8] and address barriers to participation [9, 10].

The Swedish PAP concept (FaR®) consists of motivational person-centred counselling, along with individualized written prescriptions of home-based activities (e.g. walking) and/or facility-based activities in local sports organizations and follow-up by health care professionals [11]. Some health care organizations in Sweden have implemented multi-professional programmes for delivery of PAP in routine health care involving physical activity counsellors [11]. Such programmes have been proposed as a possible means to facilitate delivery of PAP in routine health care and to improve the effectiveness of PAP to increase patients’ physical activity levels [4, 12].

The Swedish PAP concept has been favourably evaluated in terms of the effect on patients’ change in physical activity levels and quality of life [13–15], adherence to prescription [16, 17] and the characteristics of prescribed patients and prescribers [18]. Further, experiences of PAP among patients with chronic musculoskeletal pain have been described [4]. However, the concept has not been investigated from a patient perspective to gain an understanding of how routine health care delivery of PAP and counsellor support may influence the recipients’ beliefs, attitudes and motivation to achieve increased physical activity. In general, there is a paucity of qualitative research on routine health care delivery of PAP from the patient perspective, including programmes that include access to counsellor support. Such research could potentially yield important insights into PAP recipients’ paths to behaviour change, i.e. increased physical activity. This knowledge is highly relevant for the development of improved PAP programmes. To address this gap in research on PAP, this study investigates the experiences of PAP recipients in a routine health care PAP programme in Sweden that offers the recipients support from physical activity counsellors. The aim was to explore influences on PAP recipients’ engagement in physical activity from a long-term perspective.

## Methods

### Study design

A qualitative explorative design was used, operationalized through individual semi-structured interviews with a purposively selected sample of PAP recipients. A combination of conventional (inductive) and directed (deductive) approaches to qualitative content analysis was used [19, 20].

### Study setting

Interviews were conducted with PAP recipients living in the County of Kronoberg, a small rural county in the southern part of Sweden, with approximately 190,000 inhabitants. The health care organization in this county is managed by the County Council of Kronoberg and consists of 22 public and 11 privately operated primary health care centres (all publicly funded) and secondary care provided in two public hospitals.

The Swedish welfare system is largely funded by taxes and provides equal access to health care for everyone, with elderly care and social services based on each person’s need for support. The system is characterized by shared welfare responsibility between 21 county councils and 290 municipalities. County councils provide primary and secondary health care, whereas municipalities are responsible for social services and providing health care for older people living at home or in nursing homes.

In 2009–2010, the County Council of Kronoberg implemented a multi-professional programme for health care delivery of PAP in both primary and secondary care. The PAP programme features the key elements of the Swedish PAP concept [11]. The prescriptions are issued by licensed health care professionals, e.g. physicians, nurses and psychologists, during routine health care visits. In conjunction with issuing the PAP, the prescriber provides written information about the physical activity counsellors. There is no formal referral system arranged between prescribers and counsellors. PAP recipients who want this support contact a counsellor by e-mail or phone. This counselling support is free of charge and can be utilized for 1 year after the PAP is issued. The counsellors are licensed health care professionals (registered nurses and physiotherapists) who are trained in motivational interviewing (MI) counselling techniques. They are familiar with local facility-based activities. Any fees associated with participation in activities are paid for by the PAP recipients.

### Participants and recruitment

The participants in this study were purposively selected from PAP recipients in routine care between June 2013 and June 2014. The sample was selected to represent various age groups (18–29, 30–44, 45–64 and 65+ years), sex and municipality, prescribing health care setting and unit, and use of counsellor support (users and non-users). Criteria for participation were Swedish-speaking, willingness to share experiences with the researcher and having received a PAP 1.5–2.5 years earlier.

In total, 75 recipients were invited, 68 by postal invitation and 7 by telephone. Telephone invitations were used with the aim of reaching a younger and more geographically diverse sample. All participants received both written and verbal information about the study. A heterogeneous sample of 13 of the 75 invited recipients agreed to participate (Table 1).

**Table 1:** Characteristics of the PAP recipients who were interviewed

Characteristic	Number (*N* = 13)
Sex	
Female	9
Male	4
Age	
30–44 years	1
45–64 years	3
65+	9
Prescribing setting	
Primary care	11
Secondary care	2
Visits to PAP counsellor	
Yes	6
No	7
Number of inhabitants in the municipality of residence	
< 15,000	3
15,000–30,000	5
> 80,000	5

### Data collection

Data were collected from April 2015 to January 2017. A semi-structured interview guide was developed by the researchers in order to give the interviewees the opportunity and freedom to express their individual views on their engagement in physical activity. All questions were generated by the researchers informed by previous research about PAP and barriers and facilitators to physical activity behaviour.

The interview guide consisted of four main areas of questions: prescription and counselling; start of activity; maintenance of activity; and perceived benefits of PAP (see Additional file 1). A picture showing the topics was visible to the participant and the interviewer during the interviews. The interviews started with a key question for each main area followed by supplementary questions when necessary. Descriptive questions were used [21], such as “Could you tell me about the consultation when you received PAP” followed by probing questions, e.g. “Can you explain a little further?”, and/or clarifying questions, e.g. “What advice did you receive from the prescriber?” At the end of each interview, the interviewer asked if there was anything that had not been elucidated.

The interview guide was tested in one interview to determine whether the questions were suitable for obtaining rich answers. The questions were found to be clear and informative. Consequently, no revision of the interview guide was done and the test interview was included in the analysis.

All interviews were conducted by the first author (PA), either in the participant’s home or another location selected by the interviewee. A maximum of 60 min was allocated for each interview. The interviews were recorded and transcribed verbatim, mainly by the interviewer (PA). Two were transcribed by an assistant. PA listened and read through the first version of all transcriptions and made corrections where necessary.

### Theoretical framework

The interview questions were informed by the theoretical framework Capability-Opportunity-Motivation-Behaviour (COM-B) [22]. COM-B was also used as a framework to analyse the data by means of categorizing the factors (influences on the behaviour) into the three behaviour change determinants, i.e. Capability-Opportunity-Motivation, in the deductive analysis.

The COM-B framework was developed with reference to existing theories of behaviour and a US consensus meeting of behavioural theorists, which considered the prerequisites for enacting various behaviours [22]. The framework is intended to be comprehensive, parsimonious and applicable to all behaviours. COM-B has been widely applied to many different types of behaviours, including studies of audiologists’ behavioural planning [23], adherence to swallowing exercise [24] and promoters and barriers to being vaccinated and taking antiviral drugs [25].

Capability is defined in COM-B as an individual’s psychological and physical capacity to engage in the behaviour concerned, encompassing having the necessary knowledge, ability and skills. Opportunity is defined as all the factors that lie outside the individuals that make the behaviour possible or prompt it. Motivation or willingness to enact behaviour is defined as all those brain processes that energize and direct individuals’ behaviour. Motivation includes habitual processes, emotional responding, as well as conscious, deliberate decision making [22]. Behaviour in this study was defined as undertaking and maintaining some form of physical activity.

### Data analysis

The analysis was grounded in qualitative content analysis according to Krippendorf [20]. The interview transcripts were read through several times by PA and LL to obtain a sense of the whole. The text was then divided into meaning units (paragraphs) in relation to the aim (by PA and LL). The meaning units were condensed, with the purpose of reducing the text, but still preserving the core. The meaning units were then abstracted and labelled with a code. The codes were compared based on differences and similarities, and sorted into sub-categories, structured according to the predefined categories, i.e. the determinants in COM-B (by PA, LL and PN).

In the next step, the highlighted text was read, coded and categorized separately by PA, LL and PN. The text was analysed individually by the three authors to ensure credibility, using a structured process to code and categorize the data according to the COM-B framework. Then, the authors discussed the interpretation of the data in relation to COM-B and compared their coding. Discussions in the group continued until no inconsistencies existed and a shared understanding was reached to prevent researcher bias and strengthen the internal validity [26].

Representative quotations were identified to report the findings. Quotations were then translated from Swedish to English by PA and an assistant (native Swedish speaker and with good competence in verbal and written English), and thereafter controlled by PA and LL.

## Results

Analysis of the data yielded ten factors (i.e. sub-categories) that influenced the participants’ engagement in physical activity. They were mapped onto the three overarching categories of the COM-B framework. In the following, the results are presented in accordance with COM-B.

### Capability to engage in physical activity

PAP recipients’ capability to engage in physical activity was associated with adapting the PAP to the individual’s physical capacity and taking into account the individual’s previous experiences of physical activity.

### Tailoring the PAP to the individual’s physical capacity

It was important that the prescribed activities were tailored to the PAP recipient’s physical capacity. This capacity varied among the interviewees and was influenced by factors such as age, level of previous physical activity and health status, e.g. the type, severity and number of diseases. Trying out different activities provided a means to find an appropriate physical activity.

I should exercise my knees and hips just to keep them smooth, so that I could avoid surgery. If they stay like this there will be no surgery. I have no pain if I do not overwork. Female ≥65 years (4).

I went on Easyline [a specific activity] once, but it was way too hard for me. But then a softer class started; that one was not so hard, so I joined that class. Female ≥65 years (3).

If a prescribed activity was found to be ineffective in achieving desired outcomes, the interviewees emphasized that a change of activity was needed even if this meant that the new activity might be less appealing to them. Switching to another activity might also be prompted by a change in the health condition.

The prescription was that I should walk four to five times a week, I think, and then two to three exercise classes a week. But then my blood pressure was too high so my counsellor didn’t dare to advise me to join these classes, but I could start walking and doing water exercise as I always have. I’m glad my body responded so well to the activity, so that I did not have to go to a gym. Of course, I would have done that if I needed to. Female ≥65 years (5).

### Accounting for the individual’s experiences of physical activity

Previous negative experiences of physical activity, e.g. experiences of exclusion from school sports activities, contributed to a degree of resistance to physical activity in general and/or to participation in specific activities.

If it had been just an ordinary sports club, I probably would not have joined it, because I never liked gymnastics. I belong to the category that was always chosen last in school; no one wanted me on their team. Female ≥65 years (4).

I don’t like water exercise because there are a lot of people, but then I started with Easyline, which is also a group activity. I think it’s OK but I like to do my own moves. Female ≥65 years (3).

Positive experiences of the prescribed activity were important for engagement and maintenance. Interviewees talked about the importance of having fun while undertaking the physical activity, with some expressing a fondness for group activities because they allowed for socializing. The feeling of not being comfortable in an activity or a group hindered further physical activity.

They really did not have much to offer except swimming. There were just females when I got there, it was nice, but it felt strange. I don’t like water exercise and things like that. Male ≥65 years (10).

If you don’t think it’s fun, you postpone it. I’ve been involved in Friskis and Svettis [a popular Swedish training organization] but, no, I didn’t like it either. I’ve access to a gym, where I can train as much as I want, but I don’t do it either. Female ≥65 years (13).

Having previous experience of the benefits of physical activity contributed to maintaining physical activity. In contrast, having knowledge or recognizing the importance or advantages of physical activity, but lacking first-hand experience, did not seem to facilitate PAP recipients’ engagement or sustainability.

You know the importance of exercise, I have the knowledge. If I didn’t have the knowledge, maybe I could have been more curious. I have to admit I didn’t prioritize it [physical activity], but I would need to. Female 45–64 years (12).

### Opportunity to engage in physical activity

PAP recipients’ opportunity to engage in physical activity was related to receiving a prescription, receiving professional counselling and follow-up from a physical activity counsellor, collaboration between prescriber and counsellor, having access to appropriate activities, having a balanced life situation and having support from someone who can encourage continued physical activity.

### Receiving a prescription

PAP was seen as something to be taken seriously by the interviewees. Receiving a prescription for physical activity was perceived as more important for carrying out the activity than merely talking about or answering questions about one’s physical activity without a PAP. On the other hand, the written prescription in itself seemed to have only a symbolic value, as few interviewees remembered whether they had actually received a prescription on paper or not. Some interviewees had changed their physical activity over time, which meant that a paper prescription received in health care no longer applied.

When I got it on prescription, it became a little more serious. Now I have this prescription. It’s just like medicine, the doctor prescribes a pill a day, instead the doctor prescribed water exercise once a week and then you have to go. Female ≥65 years (4).

I think it’s good, it’s important with physical activity. But I think it takes more than this prescription. I think you need to be motivated as well, but it’s always good to have support. Male, 30–44 years (11).

### Receiving professional counselling and follow-up from a physical activity counsellor

All interviewees expressed that receiving counselling and follow-up were important aspects of PAP. The six interviewees who had visited a PAP counsellor expressed a great deal of confidence in the physical activity counsellors’ professional competence. Several of those interviewed were eager to increase their physical activity, but few believed they could not achieve this entirely on their own. Being followed up by a counsellor was deemed important for continuing a prescribed physical activity.

Some people tell you what you should do and that’s a negative thing. In this case it was never like that; if you had not done what you were expected to, we tried to find another way to do it. I think it’s very important that the personal chemistry is right, and it was. Female ≥65 years (13).

The duration of support varied from a couple of months to more than a year. Not having a pre-determined schedule of support facilitated change of physical activity, and even sustained activity.

[Concerning the counsellor visits:] I was there for more than a year; it was me who wanted to see her more. Male 45–64 years (1).

Dialogue with a counsellor made it easier to find a suitable physical activity that matched the PAP recipients’ interests, health condition and financial capacity. The counsellors’ knowledge about local activities was appreciated by the interviewees. Interviewees who visited a PAP counsellor received information about locally organized activities, which they often did not know about themselves. Interviewees who did not visit a counsellor often lacked information about the range of available activities.

It was the counsellor who came up with some suggestions for activities and then some estimates about how much it would cost. I choose Medley [a physical activity organization] because they had swimming and other activities that you could combine. Male, 45–64 years (1).

Ordinary table tennis and that kind of stuff is fun. You meet people and there may be some competitions and stuff like that. It’s fun, but I don’t know if there are things like that. Male ≥65 years (10).

The counsellors also introduced the PAP recipients to tools such as mobile apps, training diaries and pedometers, which were helpful for some and were mostly used periodically.

For a while I tried to use the App that the counsellor had, and for a while I tried to write down what I ate. So I was very careful for a while, but it was too advanced and took too long. Female, 45–64 years (9).

### Collaboration between prescriber and counsellor

Most of the interviewees perceived that it was difficult to make the first contact with a counsellor because this was a new function that was unknown for them. One reason for not seeking support was that the interviewees trusted in their own ability to change their activity; a high level of trust in their own ability contributed to not seeking counsellor support.

It was a bit difficult to make the first contact, because I had not recognized that there was a problem. Female ≥65 years (2).

When I accepted this offer, the nurse contacted the counsellor and then she [the counsellor] contacted me. Otherwise it’s easy to pass. Female ≥65 years (13).

The way the prescriber informed or introduced the recipients to the availability of the PAP counsellors influenced whether they used this support or not. The interviewees were of the opinion that the prescriber had to actively promote the use of counsellor support. Receiving a written leaflet was not enough to seek counsellor support.

At that time I didn’t have the power to get out. It would have had more effect if someone had called, I have to admit. Female 45–64 years (12).

I just received a paper and then I put it away; then we went down to the summer house and it petered out.. Nothing came of it. Male ≥65 years (7).

Interviewees argued that health care professionals who prescribe physical activity without following up with questions about how it went show a lack of interest in PAP, which could reduce the likelihood of sustained physical activity.

In my case, it was me who had to take the initiative completely. It feels like they write a prescription and then they do not bother anymore. Female ≥65 years (2).

[Concerning follow-ups by the prescriber:] It didn’t work out so well, to be honest. Male ≥65 years (10).

### Having access to appropriate activities

Proximity to nature allowed PAP recipients to engage in home-based activities such as cycling, walking and Nordic walking. Several of the interviewees appreciated outdoor activities, because they enjoyed seasonal fluctuations in nature, and the animal and bird life.

I can choose where I want to go for a walk and I usually go in the forest. I enjoy seeing how the seasons change. Female ≥65 years (5).

There is nothing more delightful than going for a walk and listening to the birds. Female ≥65 years (4).

Seasonal variations influenced interviewees’ engagement in and choice of physical activity. For most of the interviewees, indoor activities were most common from September to April, whereas outdoor activities were more likely during the spring and summer months. Several of the interviewees did not want to be dependent on fixed times for organized activities during the spring and summer, preferring to be more active on their own during this time. Most organized group activities take a break during Christmas and New Year as well as during the summer. Some interviewees found it hard to resume the activity after a break, as the habit was interrupted.

I don’t like to go the indoor swimming pool when it’s spring and summer. In the summer, I do more outdoors activities. Female ≥65 years (5).

Well, in the summer, you don’t get away so it drops. Female 45-64 years (9).

To go to the gym was inconceivable for some if they did not receive personalized support. Some of the interviewees who were unaccustomed to training in a gym emphasized the importance of receiving a demonstration of the machines and equipment as well as having a personal training programme put together by a gym instructor.

I got help from a personal trainer who gave me some extra exercises, if I remember correctly. It was at the gym I started. Male 30–44 years (11).

[Attending a gym without personal instruction:] No, I don’t think you would know where to start, or which machines to use. Male ≥65 years (8).

Some interviewees preferred group activities that were exclusive to PAP recipients, with the prescription being the admission ticket. Prolonged participation in these activities required a new PAP.

As long it’s a group for people with physical activity prescription who have the same problem. To go to activity centres where most people are fit does not feel good for someone who is very out of shape as I am. Female 45–64 years (12).

When talking to people, you realize that you are better off than some others. Female ≥65 years (4).

### Having a balanced life situation

Having a life situation whereby work and leisure time are balanced made it easier to engage in physical activity, according to the interviewees. They perceived a heavy workload and other factors that complicated the “life puzzle” as barriers to pursuing sustained physical activity. Receiving a PAP at a time when one’s leisure time was limited made it difficult to prioritize physical activity.

I slowed down and started a new job. I felt that I had more spare time. I could find the time because I have a pretty flexible job. But I changed my job, with more responsibility, so it has become more difficult. Male, 30–44 years (11).

### Having support and someone who can encourage continued physical activity

The interviewees believed it was important to have someone who could actively “push” them to adhere to their physical activity over time, especially when it was tough. The PAP counsellor was such a person. Family members, not least the spouse, were also described as important persons for continued physical activity. Some interviewees experienced the social support given in group-based activities as important.

Socially I have gained more contacts and met equals. I can give some good advice to others and then feel pleased with myself. Female ≥65 years (4).

We push each other a bit, the wife and me, to do this. Male ≥65 years (8).

### Motivation to engage in physical activity

PAP recipients’ motivation to engage in physical activity was associated with the individual’s own desire to improve his or her health condition and finding activities that encouraged continuation.

### The individual’s own desire to improve his or her health condition

Positive health outcomes from the physical activity were a motivational factor for all the interviewees. Achieving and experiencing various positive results were seen as highly rewarding, functioning as a strong motivator for continued activity. The PAP counsellor was described as a supportive function, which was motivational.

It feels like a reward when you go to the counsellor and see that your blood pressure is lower. Then I have one more thing that triggers me; it is an App called Run Keeper, which makes me compete against myself. Female ≥65 years (5).

When you have lived with pain for a year and then it starts to disappear. It was the biggest, coolest thing. Male, 30–44 years (11).

Engaging in physical activity to avoid disease and premature death was also a motivational factor for some of the interviewees.

Actually, it was my doctor who shocked me when my blood pressure was too high and she told me that we have to do something, you should not die now. It was drastic, but it made me react. This is serious, you have to do something, and therefore I have been very motivated. Female ≥65 years (5).

I have two older siblings who have diabetes, and I don’t want to have diabetes. I had a brother who died unexpectedly. I think his blood pressure was high. It makes you think. Then I got high blood pressure myself. Female ≥65 years (9).

### Finding activities that encourage continuation

Activities that were perceived to be stimulating or fun motivated the interviewees to sustain physical activity over a longer period of time. Some of the interviewees found this in activities that enabled them to socialize with others. In contrast, an activity perceived as not fun was a barrier for continued activity.

[Motivation for continued activity:] I think it’s fun, and we are a group; we meet once in a while. Female ≥65 years (3).

[Motivation for continued activity:] There were two machines at the gym or something like that, and it was not fun, so I paid for three months, but I think I was not there more than three times. Female ≥65 years (6).

## Discussion

This study investigated influences on PAP recipients’ engagement in physical activity. Analysis of the interview data yielded ten factors that were perceived to influence the interviewees’ engagement in physical activity in the 1.5–2.5 years after receiving the prescription. The factors were mapped onto COM-B; a framework that posits that behaviour (such as engaging in physical activity) depends on the capability, opportunity and motivation to undertake this behaviour. The three determinants of COM-B interact to influence behaviour, which in turn influences these components [22]. COM-B was found to be useful to categorize factors found to influence physical activity.

Two of the factors concerned the PAP recipients’ capability to engage in physical activity: tailoring the PAP to the individual’s physical capacity and accounting for the individual’s earlier experiences of physical activity. These factors underscore the importance of adapting the physical activity to each PAP recipient’s physical and psychological capacity. An individual prescription of physical activity is considered one of the key components in the Swedish PAP concept [5], and our findings lend credence to the importance of accounting for the individual characteristics of the recipients.

The PAP recipients’ previous experiences of physical activity seemed to influence their initiation of activity, whereas experiences during the activity period were important for sustained activity. We found that the existence of diseases influenced the PAP recipients’ capability to engage in physical activity, which is in accordance with previous research in which diseases were associated with fear and avoidance of activity [6, 27, 28]. Diseases or bodily symptoms have been found to negatively influence the self-efficacy of PAP recipients, i.e. belief in one’s capability to complete tasks and reach goals in becoming more physically active [29]. Matching the activity with the individual’s capacity and experience can be expected to enhance the individual’s self-efficacy with regard to physical activity [30]. Mastery of experiences, i.e. previous successful performance of physical activity, is an important factor determining a person’s self-efficacy, and a higher level of self-efficacy can be achieved by means of feedback on past performance [31].

Six of the ten factors influencing the PAP recipients’ physical activity were attributed to opportunity to engage in physical activity, which in COM-B refers to extraneous factors that enable or bring about behaviour. Receiving professional counselling and follow-up from a physical activity counsellor as well as having support and someone who can “push” for continued physical activity were considered important. The importance of social influences on behaviours is highlighted in many social cognitive theories on behaviour change. For example, Social Cognitive Theory [30] assumes that an individual’s behaviours are influenced by the actions that the individual has observed in others. This was evident in our study. We found that PAP recipients believed that they were influenced by being “observed” by others, most importantly the counsellors and spouses. A previous study found that spousal support for physical activity was perceived to be important for regular exercise but shared participation in physical activity was uncommon; regular exercise appeared to be largely individual and independent of others [32].

Our findings point to the importance of PAP counsellors in supporting the PAP recipients to find an appropriate physical activity and achieving sustainability of the activity over time. This is in line with previous studies of multi-professional PAP programmes, which have suggested that physical activity counsellors enhance physical activity in prescribed patients [4, 33–35]. In this study, we found that the prescribers appeared to be the deciding factor for PAP recipients to seek counsellor support because they informed the patient about the option to receive counsellor support or referred directly to a counsellor. The flexibility in the duration of counsellor support found in our study seemed to support sustained physical activity for the PAP recipients. Counsellor visits were offered based on each recipient’s personal needs, and some PAP recipients seemed to require support for more than a year after the prescription. To our knowledge, this has not been described in previous studies of PAP from the patient perspective. There is no “one-size-fits-all” programme for PAP, a finding that is supported by other PAP studies from the patient perspective [9, 29, 36–38].

Two of the factors influencing the PAP recipients’ physical activity were related to their motivation: the desire to improve his or her health condition and finding appropriate activities that encourage continuation. Both factors suggest the relevance of having more autonomous motivation, i.e. engaging in physical activity for personal interests and enjoyment, rather than doing it because one feels compelled by pressures, i.e. controlled motivation [39]. Self-Determination Theory posits that all behaviours lie along a continuum of relative autonomy, reflecting the extent to which a person endorses what he or she is doing. At one end of the continuum is behaviour that is intrinsically motivated and performed for its inherent satisfaction, e.g. for the fun, interest or challenge it offers. At the other end, is amotivation, which refers to a lack of intention to perform the behaviour. A considerable body of research exists that shows that more autonomously motivated behaviours’ are more stable, performed with greater care and quality, and accompanied by more positive experiences [40]. Studies on physical activity have confirmed the relevance of achieving more autonomous forms of motivation [41]. For example, Fortier et al. [42] found that physical activity counsellors trained in Self-Determination Theory and MI facilitated change in recipients’ physical activity behaviour by fostering both the quantity and quality of motivation.

There was a great deal of interdependency among the different factors (i.e. sub-categories) and the three COM-B determinants (i.e. categories), with many of the factors related to opportunity having an impact on PAP recipients’ capability and motivation to engage in physical activity. The success of PAP programmes depends on viewing physical activity not merely as an endeavour or task for the individual PAP recipient; physical activity must be considered in the broader context of the recipient’s social circumstances.

This study has a number of methodological shortcomings that must be considered when interpreting the results. The factors influencing PAP recipients’ engagement in physical activity should not be considered as list of all possible factors because other studies may yield different factors or prioritize different factors. The results cannot be directly transferred to other county councils or international settings. We sought analytical generalization by comparing our findings with comparable studies.

The sample was purposively selected to account for a variety of characteristics. However, we were unsuccessful in recruiting PAP recipients in the 18–29 years age range. We lack information on why invited PAP recipients declined interviews, although some of the reasons that were reported by non-participants were “I did not receive a PAP” and “I could not participate in any activity due to spousal or their own health status,” indicating that many did not want to participate because they had not (or believed they had not) undertaken sufficient physical activity to be involved in the study. The interviews were conducted 1.5–2.5 years after the PAP was received, which suggests a potential risk of recall bias, i.e. an error caused by differences in the accuracy or completeness of the participants’ recollections regarding initiation of their physical activity. We believed this period was necessary to obtain information about the participants’ engagement in physical activity because PAP strives to reach longer-term maintenance of physical activity in everyday life. However, because of the length of time between PAP and the interview, the study does not allow for detailed description of time-specific influences.

The study applied COM-B as a framework. When using a preconceived framework, there is a risk of neglecting ways of analysing the data that may provide important insights [43]. However, COM-B was found to provide a suitable framework for informing the data collection and analysing the data. One limitation with COM-B in this study is that the framework does not relate to a time perspective, which would have provided valuable information concerning the process of changing physical activity. In this study, the time perspective was addressed by asking the participants about factors associated with how they got started with an activity and how they maintained the activity. The selection of one specific theory, model or framework usually means that weight is placed on some aspects at the expense of others, thus offering only partial understanding. Regardless, we found COM-B to be broad enough to enable us to use an inductive approach. COM-B was not applied until the second phase of data analysis, which meant that the data had already been analysed inductively to arrive at the sub-categories, i.e. factors.

The study was conducted by a multidisciplinary team of researchers, which was strength of the study. When interpreting the data, this allowed for multiple perspectives and different pre-understandings concerning the issue of PAP, thus enhancing the rigor and credibility of the findings. The team consisted of two (female) nurses with experience in clinical patient work as well as work on miscellaneous research issues (PA, LL), a (female) primary care physician (SH) and an experienced (male) implementation researcher, who is an economist and organizational analyst (PN).

## Conclusions

In conclusion, PAP recipients’ engagement in physical activity was influenced by the capability, opportunity and motivation to undertake this behaviour. Numerous extraneous factors influenced capability and motivation; receiving professional support and follow-up by a physical activity counsellor were of utmost importance. The physical activity counsellors were found to be important for sustained activity because they use an individual approach to counselling and flexible follow-up adapted to each individual’s need for support.

## Additional file

Additional file 1: Interview guide. (DOCX 27 kb)

## Abbreviations

COM-B: Capability-Opportunity-Motivation-Behaviour
PAP: Physical activity on prescription
