# Supplementary material for: Patients’ experiences of physical activity on prescription with access to counsellors in routine care: a qualitative study in Sweden
Source: BMC Public Health. 2019 Feb 20;19:210. doi: 10.1186/s12889-019-6535-5 (PMC6381718; doi:10.1186/s12889-019-6535-5)
Supplement: Supplementary file 1 — Interview guide. (DOCX 27 kb) [file 12889_2019_6535_MOESM1_ESM.docx]

Additional file 1

**Interview guide**

Welcome, my name is (*name of the interviewer*)*.*You have previously received information about the study; is anything unclear or something you would like to ask about the study?

The aim of this study on physical activity on prescription (PAP) is to explore influences on recipients’ engagement in physical activity from a long-term perspective. The interview focuses on your experiences around PAP and there are no predetermined best answers. The figure shows the topics and the four main areas we want to discuss with you. *A separate page that includes the figure below will be visible to the respondent and the interviewer during the interview.*

The interview will be recorded and the transcript will be identified only by a code number. You are free to stop the interview at any time without giving a reason. Do you have any further questions before we start?

*Tell the participant that you now starting the recording and begin by saying the date and interview number. Continue with:* Please tell me about your experience of PAP ... you can start to tell me about what happened when you received PAP ... or feel free to start with any areas you want.

*At the end of the interview, ask:* Is there anything that had not been elucidated?

*End the interview with:* Thank you for taken your time to participate in the interview.

*If needed use: Probing questions to get more in-depth explanations e.g. can you explain a little further and clarifying questions if something is unclear e.g. what advice did you receive from the prescriber?*
